# Polyelectrolyte-grafted Ti_3_C_2_-MXenes stable in extreme salinity aquatic conditions for remediation of contaminated subsurface environments†

**DOI:** 10.1039/d0ra04348f

**Published:** 2020-07-09

**Authors:** Sehyeong Lim, Hyunsu Park, Jin Hyung Kim, Jeewon Yang, Chaesu Kwak, Jieun Kim, Seoung Young Ryu, Joohyung Lee

**Affiliations:** Department of Chemical Engineering, Myongji University 116 Myongji-ro, Cheoin-gu Yongin Gyeonggi-do 17058 Korea ljbro@mju.ac.kr

## Abstract

MXenes, an emerging class of two-dimensional materials, are recently gaining significant attention for numerous environmental applications owing to their superior hydrophilicity and unique surface functionalities, which are suitable for adsorptive removal of various aqueous contaminants. However, it has recently been shown that MXenes have poor colloidal stability in both synthetic or natural waters containing small amounts of salt ions, which will limit the potential uses of MXenes in remediation of subsurface environments that might sometimes contain considerable amounts of salt ions, and other relevant environmental applications. Herein, we develop Ti_3_C_2_-MXenes grafted with highly salt-resistant polyelectrolytes (PEs), MXene-*g*-PEs, which are colloidally stable in extreme salinity aquatic environments and have low adsorption to soil mineral substrates. The MXenes grafted with zwitterionic PEs are found to have superior mobility properties to those with anionic PEs, which are attributed to the anti-PE behavior of the grafted polymer brushes. The MXene-*g*-(zwitterionic) PEs show long-term colloidal stability over 6 months in American Petroleum Institute (API) brine with extreme salinity (ionic strength of 2 M with 182.2 mM Ca^2+^), and little adsorption (0.5 mg m^−2^) against α-alumina surfaces (2.3 m^2^ g^−1^). Furthermore, the MXene-*g*-PEs retained the excellent adsorption capacity for methylene blue as a model aqueous organic pollutant. The results suggest the great potential of the MXene-*g*-PEs as an aqueous pollutant scavenger for various environmental applications including the combined *ex situ*/*in situ* remediation, and other relevant subsurface applications.

## Abstract 2

Polyelectrolyte-grafted Ti_3_C_2_-MXenes display high colloidal stability and low adsorption to mineral substrates in extreme salinity aquatic media, while maintaining decent removal efficiency for aqueous organic dyes.

## Graphical abstract

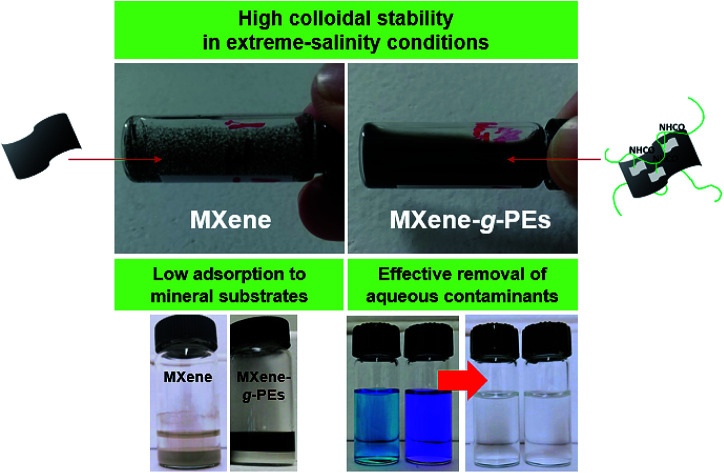

## 1. Introduction

With the rapid population growth and industrialization, aqueous contaminants originating from various sources, including dwellings, industries, transportation, and oil storage facilities, are an increasingly serious threat to ecosystems and human health. Such contaminants are difficult to manage owing to their persistence, accumulation, and lack of visibility, and enormous costs are generated in their treatment. Therefore, it is very important to secure technologies for effective and economical environmental remediation activities.^1^

Adsorption is one of the most widely used techniques for treatment of aqueous contaminants because it is easy to operate, low-cost, and environmentally friendly. So far, various nanomaterials have been considered as adsorbents, including activated carbons,^2^ silicas,^3,4^ zeolites,^5^ carbon nanotubes,^6^ and nanoscale zerovalent irons (NZVI).^7,8^ Furthermore, advanced two-dimensional (2D) materials have also been employed in numerous adsorptive water-treatment studies.^9–14^ Among these, transition metal carbides (or “MXenes”), an emerging class of 2D materials,^12–14^ have recently gained significant attention owing to their remarkable hydrophilicity, high specific surface area, and unique activated surface functionalities which could be highly beneficial for aqueous environmental remediation. In addition, MXenes have distinct advantages of high electronic conductivity, catalytic activity, and even antibacterial properties, integration of which with the fine tunability of their surface chemistry may potentially lead to generation of new types of environmental applications. For example, titanium carbide (Ti_3_C_2_)-MXenes have shown promising adsorptive performance for various aqueous pollutants including organic dyes (*e.g.*, methylene blue (MB)),^15–17^ toxic metals (*e.g.*, Cu(ii),^18,19^ Cr(vi),^20^ Hg(ii)^21^), and nuclear wastes.^22^ Owing to their 2D structure, MXenes have also been utilized to fabricate purification membranes, with which aqueous pollutants are successfully sequestrated.^23,24^ Furthermore, MXenes and their engineered derivatives have been employed in diverse environmental applications such as electrochemical separation,^25^ photocatalytic degradation of organic pollutants,^26^ and development of environmental sensors.^27^

Despite a number of previous studies, the ability of MXenes for scavenging the aquatic pollutants directly from subsurface environments has not been investigated so far. Indeed, various nanomaterials for environmental applications are nowadays being actively considered for *in situ* remediation where a pollutant scavenger is directly injected into the contaminated subsurface site, which enables active treatment of contamination sources and prevents re-contamination with relatively low costs for its operation.^28,29^

For successful realization of such types of environmental remediation, however, it is of primary importance to secure colloidal stability of the injected pollutant scavenger in an arbitrary subsurface aquatic environment,^7,8^ which may contain mono- (*e.g.*, Na^+^, K^+^), di- (*e.g.*, Ca^2+^, Mg^2+^), and other multi-valent salt ions, and sometimes has a very high ionic strength.^30,31^ In high-salinity aquatic media with high ionic strength, in particular, it is very difficult to maintain colloidal stability because of the screening of electrostatic inter-particle repulsion^7,8,30–32^ and complexation of the surface functional groups with di- and multi-valent salt ions, which may cause inter-particle bridging flocculation.^33,34^ A recent study directly showed that Ti_3_C_2_-MXenes, the most widely used species among the MXene family, easily precipitated even with minute amounts of multi-valent ions, displaying poor mobility in both synthetic or natural waters.^35^ Thus, it is anticipated that the pristine MXenes, without further engineering, will not definitely be effective in extracting aquatic pollutants from groundwater. Furthermore, the poor mobility of MXenes in high salinity media limits many potentially relevant applications such as electrical resistivity tomography for monitoring subsurface dense non-aqueous phases (DNAPL) source zone,^36,37^ or oil and gas recovery.^30–32^

Herein, we, for the first time, demonstrate the engineered Ti_3_C_2_-MXenes stable in extreme salinity aquatic environments, which would significantly broaden the application range of the MXene family. The key strategy is to chemically graft highly salt-resistant polyelectrolytes (PEs), which can remain hydrated even in extreme salinity environments, on to the silane-functionalized MXene surface. There have been a considerable number of preceding studies on preparation of polymer-grafted adsorbents for improving either adsorption capacity^38^ or colloidal stability (or “mobility” as often stated in relevant studies^8^), but studies on adsorbents grafted with salt-resistant PEs stable in high salinity media have been relatively much less focused. Obviously, there has been no study on PE grafting of MXenes for improving their colloidal stability in such challenging environments. In this study, the long-term colloidal stability of the PE-grafted MXenes, MXene-*g*-PEs, are carefully investigated in an extreme salinity environment, the American Petroleum Institute (API) brine (sodium chloride (NaCl) 8 wt% + calcium chloride (CaCl_2_) 2 wt%; ionic strength of ∼2 M and 182.2 mM Ca^2+^).^30^ Furthermore, the resistance of the MXene-*g*-PEs against undesirable adsorption to model mineral substrates in harsh environments is studied. The overall results suggest that the MXenes grafted with the zwitterionic PEs have superior mobility in high salinity media than those grafted with relatively well-known sulfonated anionic PEs,^30,39–42^ which is discussed in terms of the different brush behavior of the grafted PEs in the presence of salt ions. Finally, the ability of the MXene-*g*-PEs of scavenging a model aqueous organic pollutant, methylene blue, is shown.

## 2. Experimental

### 2.1. Materials

Ti_3_AlC_2_ was purchased from American Elements (USA). LiF was purchased from Waco (Japan). HCl, NaCl, CaCl_2_·2H_2_O, AA, sodium metabisulfite (SM), potassium persulfate (PP), and 1-ethyl-3-(3-dimethylaminopropyl)carbodiimide hydrochloride (EDC) were purchased from Daejung Co., Ltd (Korea). TEOS and APTES were purchased from Tokyo Chemical Industry Co., Ltd (Japan). Acetic acid and MB were purchased from Samchun Pure Chemical Co., Ltd (Korea). AMPS, DMAPS, and α-Al_2_O_3_ were purchased from Sigma Aldrich (USA). Lithium chloride (LiCl) and MV were purchased from Junsei Chemical Co., Ltd (Japan). Lithium hydroxide (LiOH) monohydrate was purchased from Duksan Co., Ltd (Korea).

### 2.2. Preparation of delaminated Ti_3_C_2_-MXenes

0.8 g LiF was dissolved in 10 mL of 9 M aqueous HCl under magnetic stirring for 5 min, followed by adding 0.5 g of Ti_3_AlC_2_ – the mixture was stirred at room temperature (RT) for 24 h. The product was copiously washed by DI water *via* repeated centrifugation and redispersion, until the pH of the supernatant reached >6. In the final step, the product was centrifugated at 3500 rpm for 2 min to precipitate multilayered MXenes along with residual Ti_3_AlC_2_ and obtain a dark-green aqueous dispersion of delaminated MXenes. The concentration of the as-prepared MXene dispersion was measured gravimetrically by evaporating the solvent (typically 2 mg mL^−1^).

### 2.3. Synthesis of the PEs

In the synthesis of poly(AMPS-*co*-AA) with lower *M*_w_, 10.3 g of AMPS, 1.05 g of AA, 1.62 g of PP, 1.14 g of SM, and 60 g of DI water were stirred at 80 °C for 16 h, following the procedure reported in the previous literature. In the synthesis of poly(AMPS-*co*-AA) with higher *M*_w_, 10.3 g of AMPS, 1.05 g of AA, 0.0162 g of PP, and 60 g of DI water were used following the same procedure. In the synthesis of poly(DMAPS-*co*-AA), 10.05 g of DMAPS, 1.26 g of AA, 0.036 g of PP, and 150 g of DI water were used, following the same procedure.

### 2.4. Silane treatment of MXenes

In the treatment with TEOS, 2 mL of TEOS was added to 50 mL of an aqueous MXene dispersion under vigorous stirring, and the pH was adjusted to 2 with 1 N HCl, followed by continued stirring at RT for 3 h. The product was washed three times with DI water *via* repeated centrifugation and redispersion, and the final aqueous dispersion was probe-sonicated in an ice bath for 30 min (2 s operation/1 s pause). In the treatment with APTES, 1 mL of APTES was preliminarily added to 10 mL of 5 wt% acetic acid solution, and the mixture was stirred for 15 min, followed by adding 2.5 N LiOH to adjust the pH to 8. Then, the aqueous dispersion of TEOS-treated MXene at 5 mg mL^−1^ was added, and the mixture was stirred at 65 °C for 16 h. The washing and probe-sonication of the product was performed in the same manner as in the previous step.

### 2.5. Grafting of PEs to MXenes

10 mL of APTES-treated MXene dispersion (5 mg mL^−1^, pH 5) was added to 20 mL of ∼10 wt% aqueous PE solution (pH 5) of either poly(AMPS-*co*-AA) or poly(DMAPS-*co*-AA) under vigorous stirring, and 0.6 g of EDC was added to the mixture, followed by adjusting the pH to 5.7 with LiOH and HCl. After stirring for 15 min, 12.5 mL of 20 wt% LiCl solution was added, and the mixture was stirred at RT for 24 h. The washing was performed in the same manner as in the previous steps, and the final dispersion was probe-sonicated in ice bath for 15 min (2 s operation/1 s pause).

### 2.6. Batch adsorption test with α-Al_2_O_3_ substrates

4 mL of either DI water or API brine containing MXenes or PE-grafted MXenes (at 2.5 mg mL^−1^) and 4 g of α-Al_2_O_3_ (average size ≤10 μm, BET surface area of 2.32 m^2^ g^−1^) were added in a 20 mL vial, and stirred with a magnetic stirrer at 300 rpm for 24 h. The stirrer was removed from the vial and the vial was placed on a flat surface to allow α-Al_2_O_3_particles to settle down for visual inspection of the adsorption of MXenes or PE-grafted MXenes. In the quantitative analysis for the adsorption of MXene-*g*-poly(DMAPS-*co*-AA) onto α-Al_2_O_3_ in API brine, a series of experiments were performed at concentrations of MXene-*g*-poly(DMAPS-*co*-AA) of 0.1, 0.5, 1, 1.25, and 2.5 mg mL^−1^ in 4 mL API brine with 4 g of added α-Al_2_O_3_. The equilibrium concentration of the MXene-*g*-poly(DMAPS-*co*-AA) was estimated by UV-vis spectroscopy based on the calibration curve established for the absorption peak at 320 nm, and the experimental data were fit to the Langmuir isotherm model using a nonlinear least-squares method.

### 2.7. Dye removal test

For the MB removal test in DI water, 5 mL of DI water containing either MXenes or PE-grafted MXenes was purposely contaminated with MB at 40, 48, 56, 64, 72, and 80 ppm, and was magnetically stirred at 700 rpm for 1 h. The adsorbents were removed by centrifugation at 8000 rpm for 20 min, and the supernatant was collected and syringe-filtered through a non-sterile polyethylene sulfone (PES) membrane with a pore size of 0.2 μm. The equilibrium MB concentration was then estimated by UV-vis spectroscopy based on the calibration curve established for the absorption peak at 646 nm. For the dye removal test in high-salinity solutions, 5 mg of either MB or MV was added in 50 mL of aqueous 1 M NaCl or CaCl_2_ solutions, and the solution was syringe-filtered through the 0.2 μm PES membrane to discard the undissolved dyes owing to the low solubility of the dyes in high-salinity solutions. The typical saturated dye concentration was approximately 15 ppm. Then, 6 mL of ∼0.3 M NaCl or CaCl_2_ solutions, contaminated either by MB or MV at ∼5 ppm, was prepared with added PE-grafted MXenes at 3.33 mg mL^−1^, and stirred at 700 rpm for 1 h. The centrifugation and syringe-filtration were similarly performed to remove the adsorbents and the removal of dyes from the supernatants was evaluated through visual inspection and UV-vis spectroscopy. To test the reusability of MXene-*g*-PEs as adsorbents, the adsorbed dyes were washed three times with ethanol and DI water, respectively, using centrifugation. The dye removal test was then performed under identical conditions using the washed MXene-*g*-PEs. The washing and removal test were repeated up to five times.

### 2.8. Characterization

Scanning electron microscopy (SEM, S-4800, Hitachi, Japan), X-ray diffractometry (XRD, AERIS, Malvern, UK), and Fourier transform infrared spectroscopy (FTIR, Varian 660-IR, VARIAN, USA) were used to characterize the morphology, crystalline properties, and chemical functional groups of the delaminated MXenes. X-ray photoelectron spectroscopy (XPS, Thermo Scientific K-Alpha^+^, Thermo Fischer Scientific, USA) was used to perform chemical analyses of products obtained after each reaction step to evidence the success of surface modification. Transmission electron microscopy (TEM, JEM-2100F, JEOL, Japan) was used to characterize the morphology of the PE-grafted MXenes. Dynamic light scattering (DLS) and phase analysis light scattering (PALS) were performed to measure the hydrodynamic diameter (*d*_H_) and zeta potential of the particles, respectively, using a Zetasizer ZS90 (Malvern, UK). GPC (EcoSEC HLC-8320, Tosoh, Japan) was performed to characterize the *M*_w_ of the synthesized PEs. TGA (SDTA851, Mettler Toledo, USA) was used to estimate the amounts of grafted PEs. UV-vis spectroscopy (S-3100, SCINCO, Korea) was used to characterize the particle and dye concentration.

## 3. Results and discussion

### 3.1. Development of the PE-grafted MXenes

**Fig. 1:**
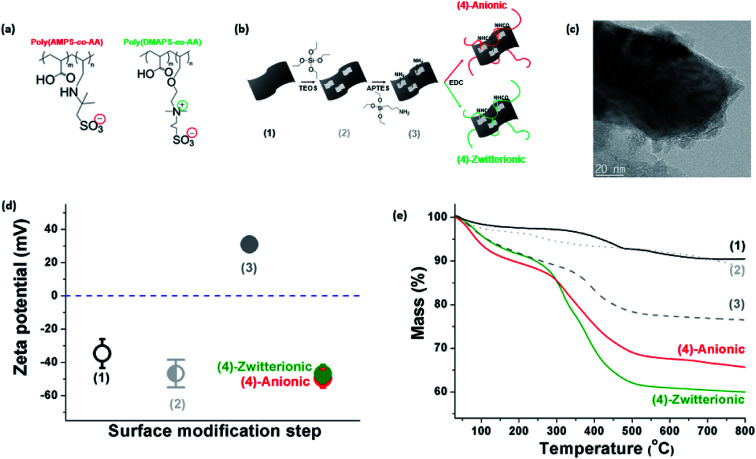
(a) Chemical structures of the synthesized copolymers. (b) Schematic diagram of the covalent PE grafting onto silane-treated MXenes. (c) Representative TEM image of the MXene-*g*-poly(DMAPS-*co*-AA). (d) Zeta potential and (e) TGA analysis at each step of the surface modification procedure.

We prepared delaminated Ti_3_C_2_-MXenes by etching titanium aluminum carbide (Ti_3_AlC_2_) with a high-concentration aqueous hydrogen chloride (HCl, 9 M) and excess lithium fluoride (LiF, 0.8 g per 0.5 g of Ti_3_AlC_2_), following a recently reported procedure.^43^ The as-prepared delaminated MXenes had excellent water dispersibility owing to the presence of hydrophilic hydroxyl surface functional groups, which are inferred from the significant absorption peaks at 3340 cm^−1^ and 1639 cm^−1^ of the FTIR spectra (Fig. S1†), and electrostatic particle repulsion, which is inferred from the measured high zeta-potential (Fig. 1).

Two types of PEs were prepared to be grafted onto the MXene surfaces. The first PE is an anionic random copolymer of 2-acrylamido-2-methylpropane sulfonic acid (AMPS) and acrylic acid (AA), poly(AMPS-*co*-AA), where AMPS is a stabilizing unit for colloidal stability in high-salinity conditions and AA is an anchoring unit supposed to condense on the MXene surface (Fig. 1a) – this anionic PE has been widely employed in a number of studies relevant to subsurface oil and gas applications.^30,39–42^ The second PE is a zwitterionic random copolymer of [2-(methacryloyloxy)ethyl]dimethyl-(3-sulfopropyl) ammonium hydroxide (DMAPS) and AA, poly(DMAPS-*co*-AA), where DMAPS is a stabilizing unit and AA is an anchoring unit (Fig. 1a). The as-synthesized PEs were characterized with gel permeation chromatography (GPC) and the weight-average molecular weight (*M*_w_) is presented in Table 1.

**Table 1:** Weight-average molecular weight (*M*_w_) of the as-synthesized PEs

Polyelectrolyte	*M*_w_
Poly(AMPS-*co*-AA)	77 024 g mol^−1^
Poly(DMAPS-*co*-AA)	123 823 g mol^−1^

**Fig. 2:**
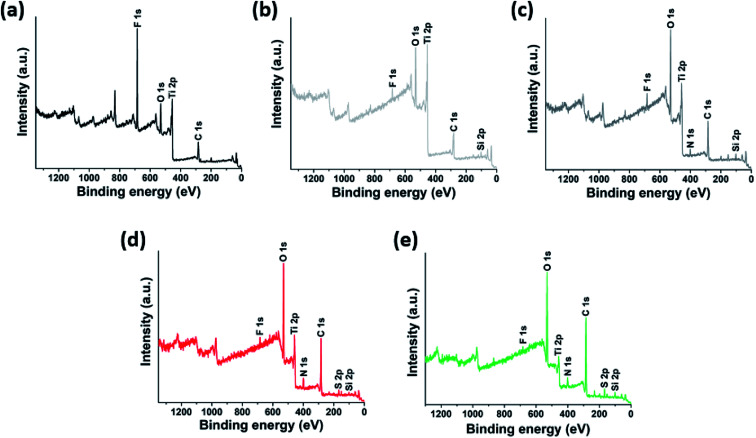
XPS survey spectra of the products obtained at each surface modification step: (a) MXene ((1)), (b) TEOS-treated MXene ((2)), (c) APTES-treated MXene ((3)), (d) MXene-*g*-poly(AMPS-*co*-AA) ((4)-anionic), (e) MXene-*g*-poly(DMAPS-*co*-AA) ((4)-zwitterionic).

**Fig. 3:**
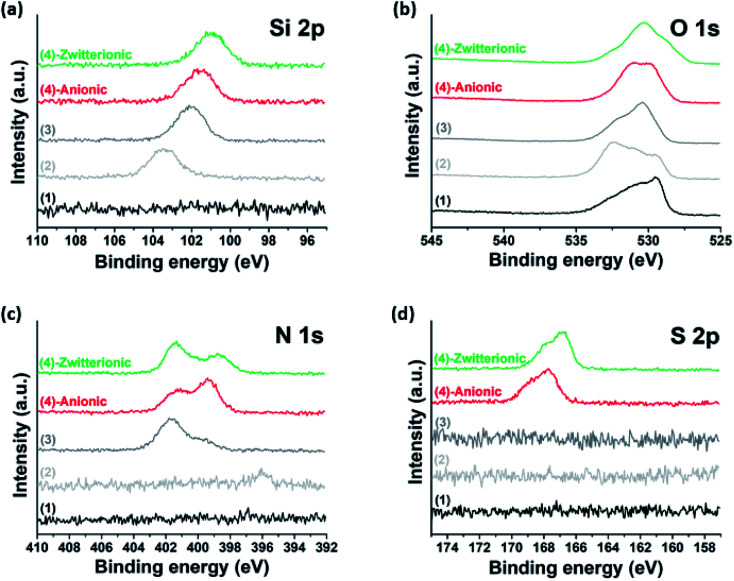
XPS narrow scan spectra for (a) Si 2p, (b) O 1s, (c) N 1s, and (d) S 2p of the products obtained at each surface modification step: MXene ((1), black), TEOS-treated MXene ((2), light grey), APTES-treated MXene ((3), grey), MXene-*g*-poly(AMPS-*co*-AA) ((4)-anionic, red), and MXene-*g*-poly(DMAPS-*co*-AA) ((4)-zwitterionic, green).

To achieve covalent grafting of PEs, the MXenes were treated with tetraethyl orthosilicate (TEOS), and subsequently with (3-aminopropyl)triethoxysilane (APTES).^30,44^ The pre-treatment with TEOS was expected to form siloxanes covalently bonded to the oxygen-bearing surface sites of the MXenes,^44^ facilitating the condensation of APTES molecules in the siloxane-to-surface direction rather than the electrostatic binding of APTES in the amine-to-surface direction which would be disadvantageous for anchoring of the PEs. After treatment with TEOS, the XPS spectra obtained for the product showed a characteristic Si 2p photoelectron peak at a binding energy of 103.4 eV (Fig. 2b and 3a) which was not observed in the untreated MXenes (Fig. 2a and 3a). In addition, a significant shift in the O 1s peak to higher binding energy was observed (Fig. 3b), suggesting the condensation of silane molecules onto oxygen-bearing surface sites of the MXenes.^44,45^ After treatment with APTES, a distinct N 1s peak was observed at 401.64 eV, which was attributed to the amine group of APTES (Fig. 2c and 3c). Furthermore, the sign for the zeta potential of MXenes was inverted from negative to positive (Fig. 1d), which confirms that the amine surface functional groups were successfully implanted on the MXene surface. Then, a multi-point covalent grafting^41^ of the AA groups of the two PEs onto the amine groups of the MXene surface was achieved through the economical carbodiimide coupling chemistry, as represented in Fig. 1b, resulting in the MXene-*g*-poly(AMPS-*co*-AA) and MXene-*g*-poly(DMAPS-*co*-AA). A representative TEM image (Fig. 1c) indicates that the product maintained the flake-like structure. The XPS spectra showed characteristic S 2p peaks at 167.83 eV for MXene-*g*-poly(AMPS-*co*-AA) (Fig. 2d and 3d) and at 166.94 eV for MXene-*g*-poly(DMAPS-*co*-AA) (Fig. 2e and 3d), respectively, indicating the presence of sulfur element, which likely originated from the grafted PEs, in both products. Also, two distinct N 1s peaks were observed for both products, where the peaks of lower binding energies, *i.e.*, 398.32 eV for MXene-*g*-poly(AMPS-*co*-AA) and 397.86 eV for MXene-*g*-poly(DMAPS-*co*-AA) (Fig. 3c), might indicate the formation of amide bond^46^ between the carboxyl anchoring groups of PEs and amine groups on silane-treated MXene surfaces. As presented in Fig. 1d, the sign for the zeta potential of MXene-*g*-PEs was inverted from positive back to negative, suggesting successful PE coating on the aminated MXenes. Thermogravimetric analysis (TGA, Fig. 1e) indicates PE contents of ∼14.15 wt% for MXene-*g*-poly(AMPS-*co*-AA) and ∼21.58 wt% for MXene-*g*-poly(DMAPS-*co*-AA). The ratio of the grafted mass of poly(DMAPS-*co*-AA) per gram of MXene (*m*_poly(DMAPS-*co*-AA)_/*m*_MXene_) relative to the grafted mass of poly(AMPS-*co*-AA) per gram of MXene (*m*_poly(AMPS-*co*-AA)_/*m*_MXene_), *i.e.*, *m*_poly(DMAPS-*co*-AA)_/*m*_poly(AMPS-*co*-AA)_ ∼1.67, is approximately consistent with the ratio of the *M*_w_ of the poly(DMAPS-*co*-AA) to that of the poly(AMPS-*co*-AA), *i.e.*, *M*_w,poly(DMAPS-*co*-AA)_/*M*_w,poly(AMPS-*co*-AA)_ ∼1.6, indicating that a similar number of PE chains were grafted on the MXene surface.

### 3.2. Colloidal stability in the extreme salinity condition

**Fig. 4:**
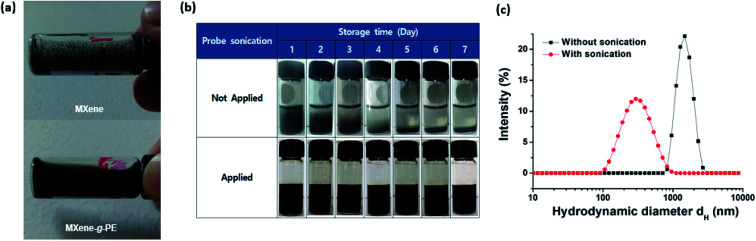
(a) Dispersion state of the pristine MXenes (upper) and the MXene-*g*-poly(AMPS-*co*-AA) (lower) in API brine. (b) MXene-*g*-poly(AMPS-*co*-AA) in API brine, prepared with (upper) or without (lower) applied probe-sonication. (c) Hydrodynamic diameter distribution of the MXene-*g*-poly(AMPS-*co*-AA) prepared with (red) or without (black) applied probe-sonication.

Fig. 4a shows the dispersion state of the unmodified pristine MXenes and the MXene-*g*-poly(AMPS-*co*-AA) in the extreme-salinity API brine (sodium chloride (NaCl) 8 wt% + calcium chloride (CaCl_2_) 2 wt%).^30^ While the pristine MXenes aggregated, formed macroscopic clumps, and precipitated immediately in the API brine, the MXene-*g*-poly(AMPS-*co*-AA) displayed excellent dispersion with no visible aggregation, which is attributed to the steric effects caused by the grafted PE chains. However, the PE-grafted MXenes also precipitated under a gravitational field within a few days (Fig. 4b) because they had a hydrodynamic diameter (*d*_H_) as large as a few microns (Fig. 4c). Although the precipitation of MXene-*g*-PEs can be readily restored by simple handshaking, and thus, it is characteristically different from the rapid precipitation of the pristine MXenes caused by their irreversible aggregation, it may still be undesirable for achieving high mobility in the subsurface field application. To overcome this problem, we probe-sonicated the MXene-*g*-PEs and reduced the *d*_H_ down to the submicron level (Fig. 4c). As a result, the sedimentation rate was significantly lowered, in such manner that no evidence of macroscopic phase separation was observed by the naked eye for a few weeks (Fig. 4b). Although the larger size MXene flakes are commonly considered superior for other types of applications (*e.g.*, electronics), it is anticipated that the pulverized nanosized MXenes, with the grafted PEs, would be more suitable for subsurface applications given its higher colloidal stability. Similar pulverization effects were also found for the MXene-*g*-poly(DMAPS-*co*-AA), as represented in Fig. S2.†

**Fig. 5:**
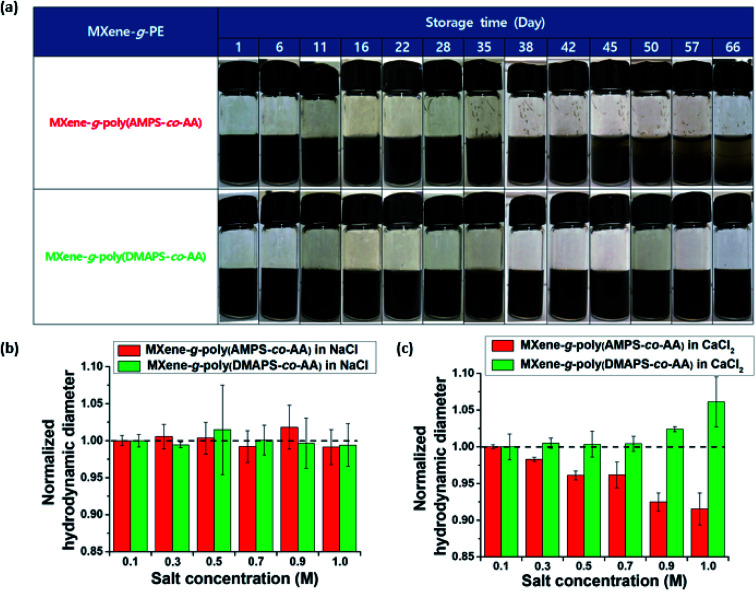
(a) Comparison of the long-term colloidal stability of the MXene-*g*-poly(AMPS-*co*-AA) (upper) and MXene-*g*-poly(DMAPS-*co*-AA) (lower) in an API brine. The *d*_H_ of the MXene-*g*-poly(AMPS-*co*-AA) and MXene-*g*-poly(DMAPS-*co*-AA) within 0.1 M ≤ *C*_s_ ≤ 1.0 M, normalized by the *d*_H_ at *C*_s_ = 0.1 M in the (b) NaCl and (c) CaCl_2_ solutions.

Fig. 5a compares long-term colloidal stability of the probe-sonicated MXene-*g*-poly(AMPS-*co*-AA) (*d*_H_ = 214.4 ± 7.8 nm at *t* = 0) and the MXene-*g*-poly(DMAPS-*co*-AA) (*d*_H_ = 234.0 ± 6.9 nm at *t* = 0) observed over the course of two months. Although both particles were colloidally stable for more than a month, a visible phase separation occurred for the MXene-*g*-poly(AMPS-*co*-AA) at day 38 which continued to result in complete sedimentation after approximately 2 months. On the other hand, the MXene-*g*-poly(DMAPS-*co*-AA) showed no visible sedimentation for more than 6 months (Fig. S3†). The difference in the sedimentation stability for both particles may be attributed to the difference in the behavior of the grafted anionic and zwitterionic PE chains in the high-salinity aquatic environment. Typically, PEs with identical sign for the chargeable unit, either polyanions or polycations, tend to contract with increasing ionic strength of the solvent due to the charge screening effects and the decrease in the osmotic pressure caused by the counterions.^32,47–49^ Furthermore, di- or multi-valent counterions undergo complexation with such PEs, causing dehydration and further contraction of the PEs.^32–34,50^ Some anionic PEs consisting of “weak” acid groups, such as poly(AA), have high binding affinity for divalent counterions, and cannot act as an effective steric colloidal stabilizer in the high-salinity environments relevant to subsurface applications (*e.g.*, API brine).^51–53^ In contrast, AMPS has a “strong” acid sulfonic (SO_3_^−^) group which is ionized in the entire pH range and has much lower binding affinity for divalent counterions.^32,54^ The complexation with divalent counterions is further prevented thermodynamically by the presence of the hydrophilic amide group.^55,56^ Because of this, PEs containing the AMPS unit have been widely employed for various subsurface oil and gas applications in high-salinity environments.^30,39–42^ However, this does not mean that the AMPS unit is perfectly free from any specific complexation with divalent counterions. For example,^32^ it was recently shown that monodisperse poly(AMPS) brushes end-grafted on silica nanoparticles underwent charge inversion by Ca^2+^ binding with excess Ca^2+^ and even the temperature-dependent “second brush collapse,” which describes the significant brush re-contraction that occurred after the brushes preliminarily underwent the “first brush collapse” regime caused by the charge neutralization at relatively lower Ca^2+^ concentrations. These findings strongly suggested the possibility of specific interaction between poly(AMPS) and Ca^2+^ ions. In contrast, the zwitterionic PEs could osmotically swell at increased ionic strength accompanying the screening of the intra- and/or inter-chain electrostatic attraction between the oppositely charged PE groups, which could increase the positive excluded volume effects of the PEs – this is referred to as the “anti-polyelectrolyte” effect.^31^ Fig. S4† shows the phase behavior of ungrafted poly(AMPS-*co*-AA) and poly(DMAPS-*co*-AA) in NaCl and CaCl_2_ solutions at high salt concentrations (*C*_s_), at *C*_s_ = 1.0 M and 4.0 M. In the NaCl solutions, both polymers were soluble up to *C*_s_ = 4.0 M. In the CaCl_2_ solutions, however, poly(AMPS-*co*-AA) showed poor solubility at *C*_s_ = 4.0 M, whereas poly(DMAPS-*co*-AA) remained soluble, indicating that the zwitterionic poly(DMAPS-*co*-AA) had higher resistance to the divalent Ca^2+^. Fig. 5b and c show the *d*_H_ of the MXene-*g*-poly(AMPS-*co*-AA) and the MXene-*g*-poly(DMAPS-*co*-AA) in NaCl and CaCl_2_ solutions within 0.1 M ≤ *C*_s_ ≤ 1.0 M, which was normalized by the *d*_H_ at *C*_s_ = 0.1 M. In the NaCl solutions (Fig. 5b), no significant variation in the measured *d*_H_ was observed with increasing *C*_s_, suggesting that the grafted brushes maintained their hydrated state at *C*_s_ = 0.1 M. In the CaCl_2_ solutions (Fig. 5c), however, the *d*_H_ of the MXene-*g*-poly(AMPS-*co*-AA) decreased gradually with the increasing *C*_s_, which reflected the contraction of the grafted poly(AMPS-*co*-AA) brushes, whereas the *d*_H_ of the MXene-*g*-poly(DMAPS-*co*-AA) increased, which is attributed to the swelling of the grafted poly(DMAPS-*co*-AA) brushes. Thus, the results suggest the importance of specific PE–Ca^2+^ interaction for the sedimentation stability of the PE-grafted MXenes in the API brine. The grafted poly(AMPS-*co*-AA) chains, which contracted with increasing Ca^2+^, likely formed a more compact brush layer with higher effective density than the poly(DMAPS-*co*-AA) chains, which swelled and had a higher excluded volume with increasing Ca^2+^. This explains the relatively faster sedimentation of the MXene-*g*-poly(AMPS-*co*-AA) than the MXene-*g*-poly(DMAPS-*co*-AA) under the gravitational field.

One may argue that the difference in the sedimentation stability shown in Fig. 5a might simply be caused by the difference in the *M*_w_ of both polymers, not by the difference in the mechanistic behavior (*i.e.*, swelling – contraction) of the grafted anionic and zwitterionic PEs in high-salinity environments. In other words, the poly(DMAPS-*co*-AA) had ∼1.6 times higher *M*_w_ (123 823 g mol^−1^) than the poly(AMPS-*co*-AA) (*M*_w_ = 77 024 g mol^−1^), which could achieve higher steric repulsion between the particles and prevent aggregation-induced sedimentation. To answer this question, we prepared a new poly(AMPS-*co*-AA) with *M*_w_ of 1 219 631 g mol^−1^, which is ∼9.8 times higher than the *M*_w_ of poly(DMAPS-*co*-AA), grafted it onto the MXene, and observed the long-term colloidal stability in the API brine (Fig. S5†). However, a visible phase separation by the particle sedimentation occurred even earlier, within 30 days, suggesting that the MXene-*g*-poly(AMPS-*co*-AA) with grafted chains of the highest *M*_w_ had the lowest colloidal stability in the API brine. Indeed, the results further support the possible effects of PE–Ca^2+^ interaction on the stability of the PE-grafted MXenes. The poly(AMPS-*co*-AA) with higher *M*_w_, containing more anionic sites, likely underwent more significant complexation with excess Ca^2+^, dehydration, and even inter-particle bridging, all of which could accelerate the particle sedimentation. Overall, the results strongly suggest that the superior colloidal stability of the MXene-*g*-poly(DMAP-*co*-AA) is due to the anti-polyelectrolyte effect of the grafted PE chains in the API brines containing the divalent Ca^2+^ ions, not due to any *M*_w_ effect.

### 3.3. Adsorption properties on mineral substrates

**Fig. 6:**
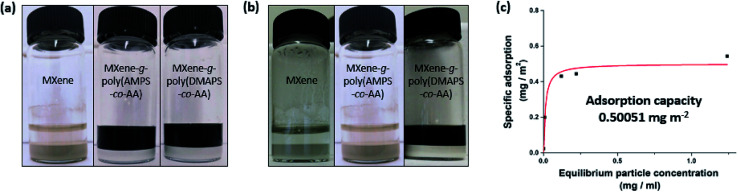
Adsorption of the pristine MXene, MXene-*g*-poly(AMPS-*co*-AA), and MXene-*g*-poly(DMAPS-*co*-AA) (from left to right) onto 4 g of ≤10 μm α-Al_2_O_3_ (BET surface area of 2.32 m^2^ g^−1^) in (a) DI water and (b) API brine. (c) Quantitative analysis for adsorption of the MXene-*g*-poly(DMAPS-*co*-AA) onto α-Al_2_O_3_ in API brine based on Langmuir isotherm model.

In addition to securing colloidal stability, it may be equally important to prevent the undesirable adsorption of pollutant scavengers onto porous soil mineral substrates with high specific surface area for high particle mobility in subsurface aquatic environment. We conducted a batch adsorption experiment where either the unmodified pristine MXenes or the PE-grafted MXenes, as an adsorbate, was suspended and agitated for 24 h in either deionized (DI) water or the API brine in the presence of added alpha-alumina (α-Al_2_O_3_) powders, as adsorbent substrates, with an average diameter of ≤ 10 μm and a BET surface area of 2.32 m^2^ g^−1^. Fig. 6 shows that the pristine MXenes completely adsorbed onto the mineral substrates in both DI water and API brine, leaving the solution phase visibly clear. In contrast, both PE-grafted MXenes remained in DI water phase (Fig. 6a), not being completely adsorbed onto the mineral substrates, which may be attributed to the (electro)steric hindrance by the grafted PEs.^7,8,30^ Interestingly, a stark contrast in the adsorption behavior was observed for the MXenes grafted by anionic and zwitterionic PEs in API brine: whereas the anionic MXene-*g*-poly(AMPS-*co*-AA), with the grafted PEs of both high (Fig. S6†) and low *M*_w_ (Fig. 6b), completely adsorbed onto the mineral substrates, the zwitterionic MXene-*g*-poly(DMAPS-*co*-AA) showed relatively higher resistance to mineral adsorption (Fig. 6b). The higher resistance to mineral adsorption of the MXene-*g*-poly(DMAPS-*co*-AA) in API brine is qualitatively correlated to their higher colloidal stability than that of the MXene-*g*-poly(AMPS-*co*-AA) in the same environment. The osmotically swollen poly(DMAPS-*co*-AA) in high-salinity environments likely remained more hydrated on the MXene surface and exerted higher positive excluded volume effects, leaving the adsorption of the PE-grafted MXenes to substrates thermodynamically less favorable. On the other hand, the collapsed poly(AMPS-*co*-AA) chains were more lyophobic, having the PE-grafted MXenes less resistant to mineral adsorption. Furthermore, the mineral adsorption could possibly be accelerated by Ca^2+^-mediated bridging between the anionic sites of the grafted PEs and the hydroxyl (–O^−^) groups on the α-Al_2_O_3_. We also performed the adsorption experiments for a range of particle concentrations (Fig. 6c) – The maximum adsorption of the MXene-*g*-poly(DMAPS-*co*-AA) per unit surface area of the α-Al_2_O_3_ in API brine was estimated to be ∼0.5 mg m^−2^ by fitting the experimental data to the Langmuir isotherm model, which is relatively low.^30^ Overall, the MXenes grafted by the zwitterionic PEs displayed superior colloidal stability and resistance to undesirable mineral adsorption in extreme-salinity conditions, which are expected to be more effective than the MXenes grafted by the anionic PEs in various subsurface applications. We also attempted to achieve simple physical grafting of the poly(DMAPS-*co*-AA) on the MXene surface through hydrogen bonding between the PE anchor groups and the MXene surface functionality, avoiding the silane pre-treatment and carbodiimide coupling reaction required for chemical (covalent) grafting. However, the product showed poor resistance to the mineral adsorption (Fig. S7†), because the weakly adsorbed PEs were likely susceptible to desorption while the particles were vigorously agitated in the presence of mineral substrates with a large specific surface area. This control experiment reveals the importance of achieving robust covalent PE grafting for successful subsurface application of the MXenes.

### 3.4. Removal of aqueous contaminants

**Fig. 7:**
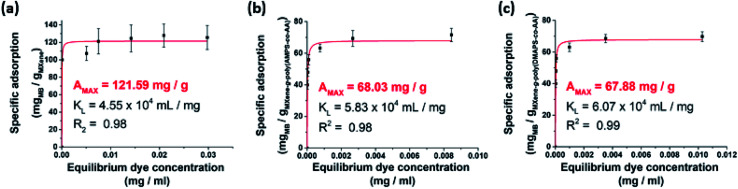
Quantitative analysis for adsorption of MB onto (a) the pristine MXene, (b) MXene-*g*-poly(AMPS-*co*-AA), and (c) MXene-*g*-poly(DMAPS-*co*-AA) in DI water.

It is important that grafting of PE should not completely deactivate the ability of MXenes to adsorb aquatic pollutants. We thus examined the removal efficiency of the PE-grafted MXenes for aqueous pollutants, where aqueous organic dyes were employed for the evaluation. We preliminarily performed a batch experiment where methylene blue (MB), which has been previously employed for studying the adsorption capacity of multi-layered MXenes,^15–17^ was an adsorbate and the unmodified pristine MXene was adsorbent. Based on the Langmuir isotherm analysis, the adsorption capacity of the delaminated MXenes for MB was found to be 121.59 mg g^−1^ (Fig. 7a), which is higher than 38.85 mg g^−1^ (ref. 15) and 99.9 mg g^−1^ (ref. 16) reported for multilayered MXenes in the previous studies. After PE grafting, the adsorption capacity was reduced to 68.04 mg g^−1^ for the MXene-*g*-poly(AMPS-*co*-AA) (Fig. 7b) and 67.88 mg g^−1^ for the MXene-*g*-poly(DMAPS-*co*-AA) (Fig. 7c), which is not surprising because many active surface sites of the pristine MXenes for dye adsorption were possibly consumed for condensation of the silane coupling agents. Nonetheless, the PE-grafted MXenes still showed decent adsorption capacities for aqueous MB removal, which are comparable to those of untreated multilayered MXenes.^15,16^

**Fig. 8:**
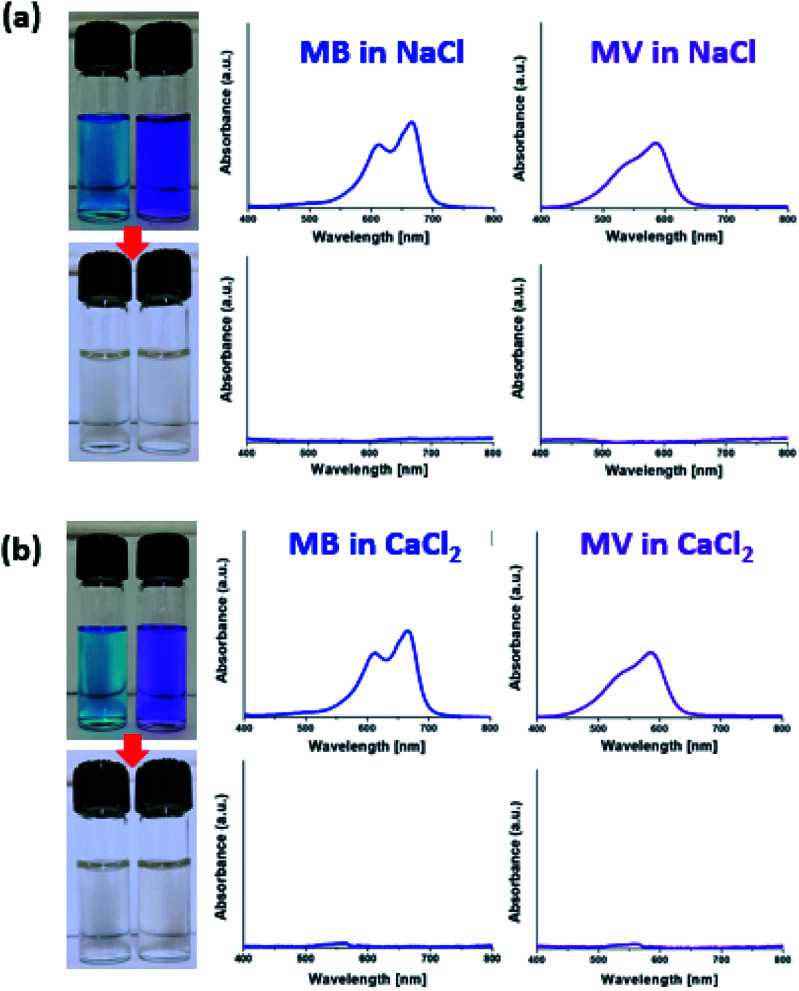
The removal of ∼5 ppm of methylene blue (MB) or methyl violet (MV) by the MXene-*g*-poly(DMAPS-*co*-AA) in (a) ∼0.3 M NaCl and (b) ∼0.3 M CaCl_2_ solutions, demonstrated by photo images and UV-vis spectroscopy.

Fig. 8 demonstrates complete removal of MB or methyl violet (MV), which was dissolved in a ∼0.3 M NaCl or CaCl_2_ solution at ∼5 ppm, by using MXene-*g*-poly(DMAPS-*co*-AA). Similar results were demonstrated for the case of using MXene-*g*-poly(AMPS-*co*-AA) in Fig. S8.† There was no systematic pH dependence of the dye removal efficiencies by both types of the MXene-*g*-PEs for similarly contaminated solutions with different pH values in the range of 2–12, as shown in Fig. S9.† We tested the reusability of two types of MXene-*g*-PEs as adsorbents, by washing the adsorbed dyes three times with ethanol and DI water, respectively, and performing the dye removal test under identical conditions. As shown in Fig. S10,† the dye removal efficiencies decreased by approximately 10% after the five recycles, but still retained the high values around 90%.

## 4. Conclusion

In this study, we engineered the Ti_3_C_2_-MXene to confer high colloidal stability and low adsorption to mineral substrates in extreme salinity aquatic environments, *via* the grafting of salt-resistant PEs on to MXene surfaces. The MXenes grafted with zwitterionic PEs showed long-term colloidal stability over 6 months in API brine with extreme salinity (ionic strength of 2 M with 182.2 mM Ca^2+^), and low adsorption (0.5 mg m^−2^) against α-alumina surfaces (2.3 m^2^ g^−1^) in the batch adsorption test. On the other hand, those grafted with well-known sulfonated anionic PEs settled down within a couple of months in API brine and were lost completely during the batch adsorption test conducted under the identical conditions. The superior mobility performances of the MXenes grafted with zwitterionic PEs were attributed to the anti-PE effect of the grafted PE brushes. The PE-grafted MXenes retained satisfactory adsorption capacity of ∼68 mg g^−1^ for methylene blue as a model aqueous organic pollutant, which is comparable to those of conventional nano adsorbents. Overall, the results suggest the great potential of the developed material as a candidate for versatile envrionmental applications ranging from the combined *ex situ*/*in situ* remediation,^37,57^ with the MXene-*g*-PEs as a pollutant scavenger, potentially to imaging of subsurface contamination source zone^37^ in conjunction with the high electronic conductivity of the base material.^12^ Furthermore, the proven colloidal stability in the testing medium (API brine) often employed in the studies on subsurface oil and gas applications strongly suggests new possibilities for using the MXenes, with the grafted PEs, as an additive of nanofluids for down-hole applicsations such as enhanced oil recovery (EOR) and reservoir characterization.^30–32,39–42^

## Conflicts of interest

The authors declare no competing financial interest.

## Supplementary Material

RA-010-D0RA04348F-s001
