# Supplementary material for: Polyelectrolyte-grafted Ti3C2-MXenes stable in extreme salinity aquatic conditions for remediation of contaminated subsurface environments
Source: RSC Adv. 2020 Jul 9;10(43):25966–78. doi: 10.1039/d0ra04348f (PMC9055327; doi:10.1039/d0ra04348f)
Supplement: RA-010-D0RA04348F-s001 [file RA-010-D0RA04348F-s001.pdf]

Electronic Supplementary Material (ESI) for RSC Advances.
This journal is © The Royal Society of Chemistry 2020

Polyelectrolyte-grafted Ti_3C_2-MXenes stable in extreme salinity aquatic conditions for remediation of contaminated subsurface environments

(Electronic Supplementary Information)

*Sehyeong Lim, Hyunsu Park, Jin Hyung Kim, Jeewon Yang, Chaesu Kwak, Jieun Kim, Seoung Young Ryu, and Joohyung Lee**

Department of Chemical Engineering, Myongji University, 116 Myongji-ro, Cheoin-gu, Yongin, Gyeonggi-do, 17058, Korea

Corresponding Author

*Email: ljbro@mju.ac.kr

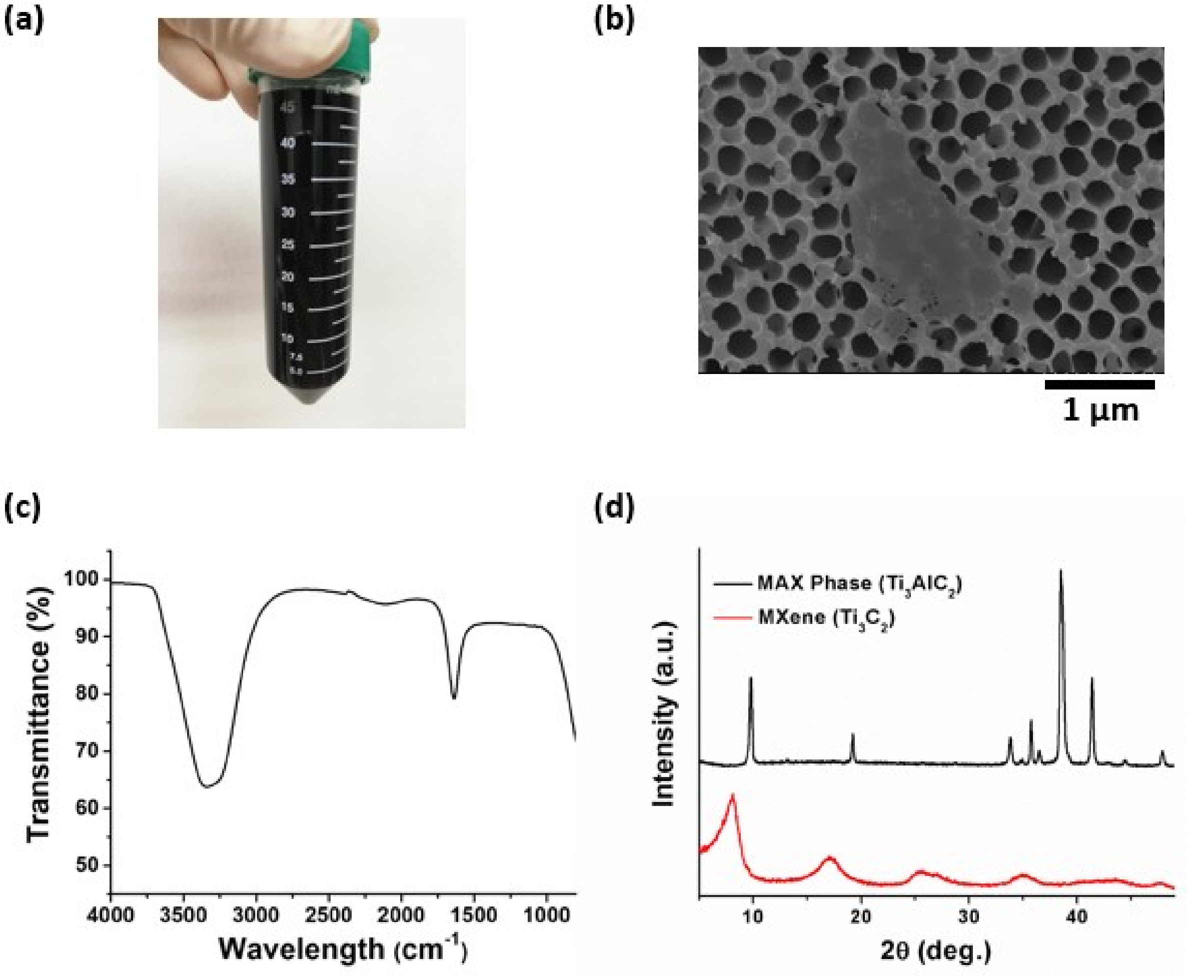

Figure S1. (a) The aqueous dispersion of delaminated Ti_3C_2-MXenes. (b) SEM image and (c) FTIR spectra of delaminated Ti_3C_2-MXenes. (d) XRD spectra of Ti_3AlC_2-MAX phase and delaminated Ti_3C_2-MXenes.

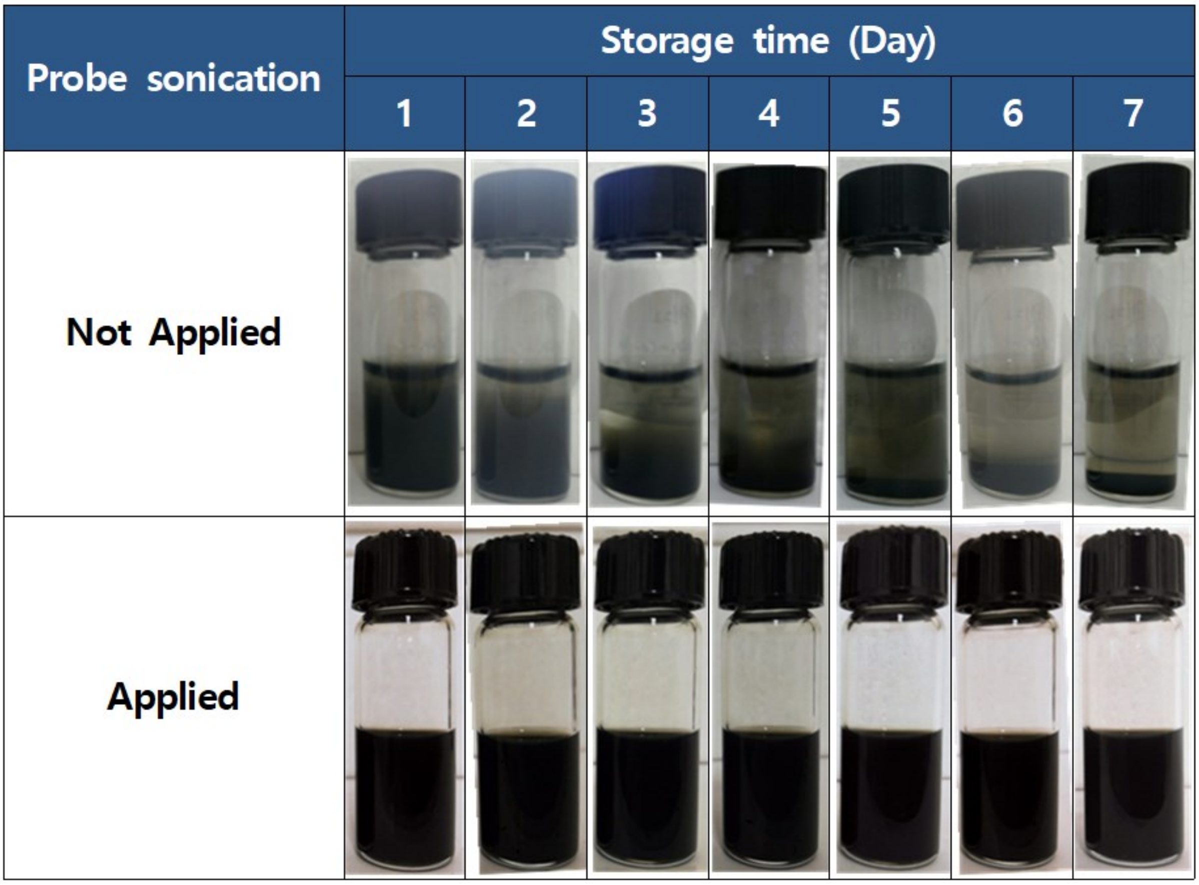

Figure S2. The MXene-*g*-poly(DMAPS-*co*-AA) in API brine, prepared with (lower) or without (upper) applied probe-sonication.

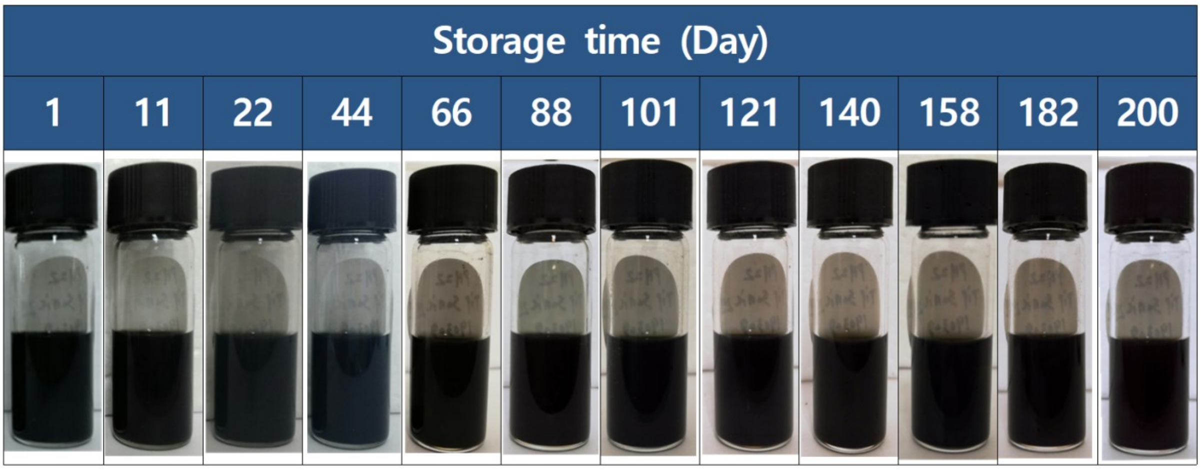

Figure S3. Long-term colloidal stability of the MXene-*g*-poly(DMAPS-*co*-AA) in API brine.

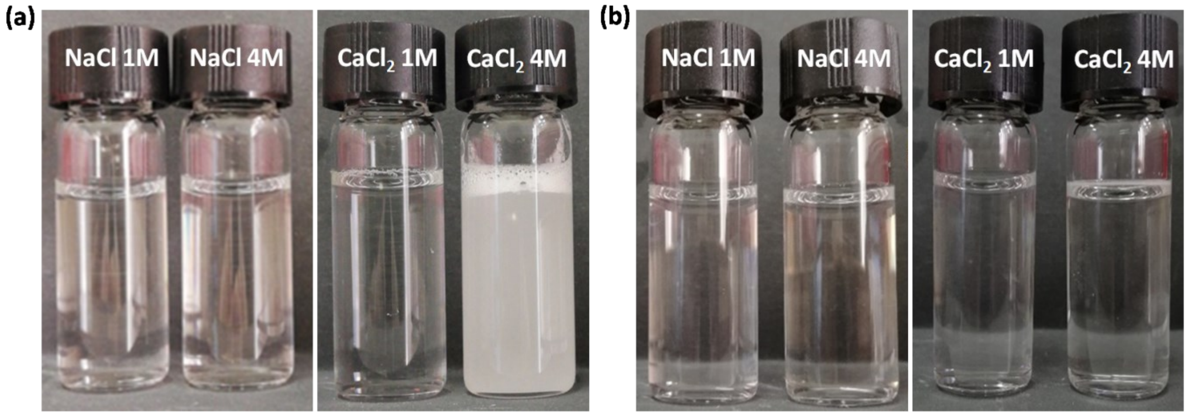

Figure S4. Phase behavior of the (a) poly(AMPS-*co*-AA) and (b) poly(DMAPS-*co*-AA) in high-salinity NaCl and $CaCl_2$ solutions, at the salt concentration (C_s) of C_s = 1 M and 4 M.

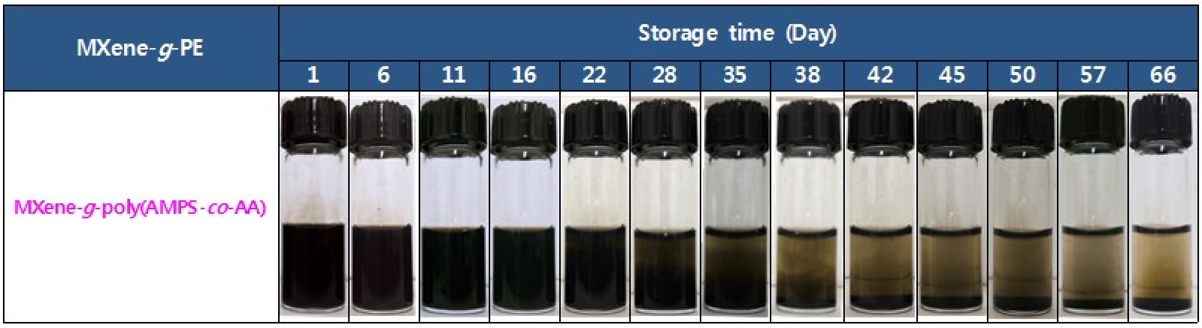

Figure S5. Long-term colloidal stability of the MXene-*g*-poly(AMPS-*co*-AA), with the grafted poly(AMPS-*co*-AA) of a high M_w (1,219,631 g mol^{-1}) in API brine.

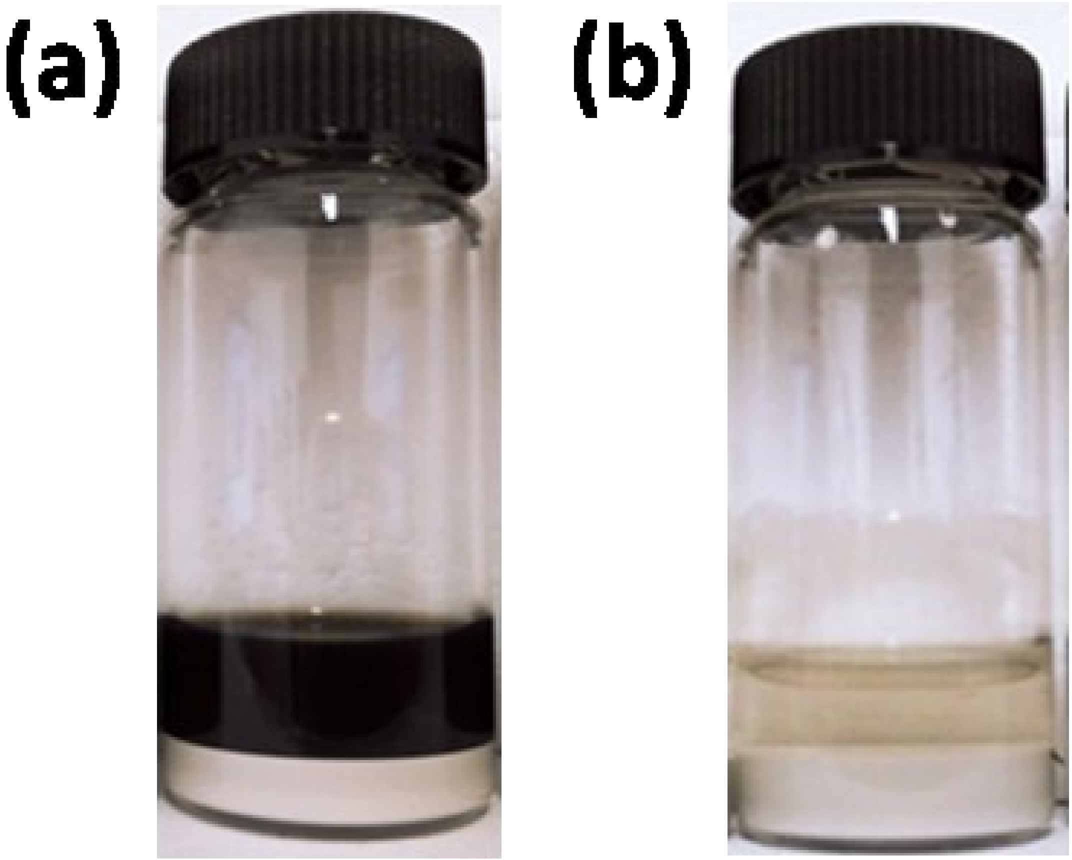

Figure S6. Adsorption of the MXene-*g*-poly(AMPS-*co*-AA), with the grafted poly(AMPS-*co*-AA) of a high M_w (1,219,631 g mol^{-1}) onto 4 g of ≤ 10 µm α-Al_2O_3 (BET surface area of 2.32 m^2 g^{-1}) in (a) DI water and (b) API brine.

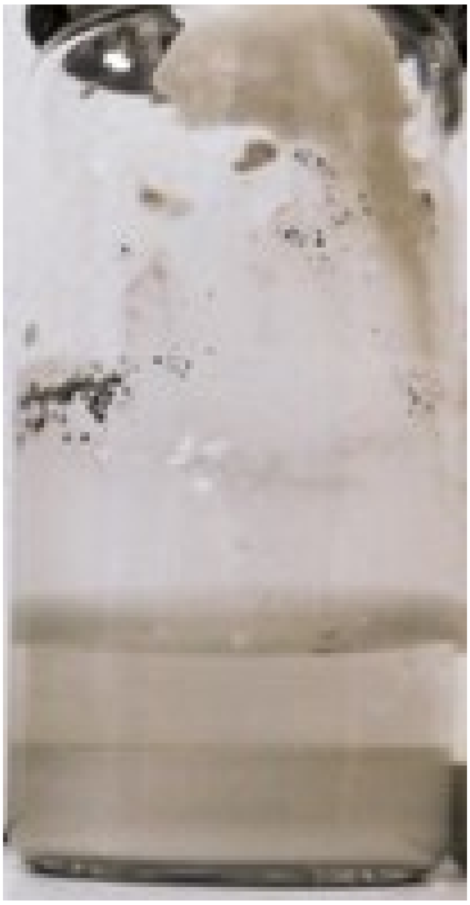

Figure S7. Adsorption of the MXenes physically (*not* covalently) grafted with poly(DMAPS-*co*-AA) onto 4 g of ≤ 10μm α-Al_2O_3 (BET surface area of 2.32 m^2 g^{-1}) in API brine.

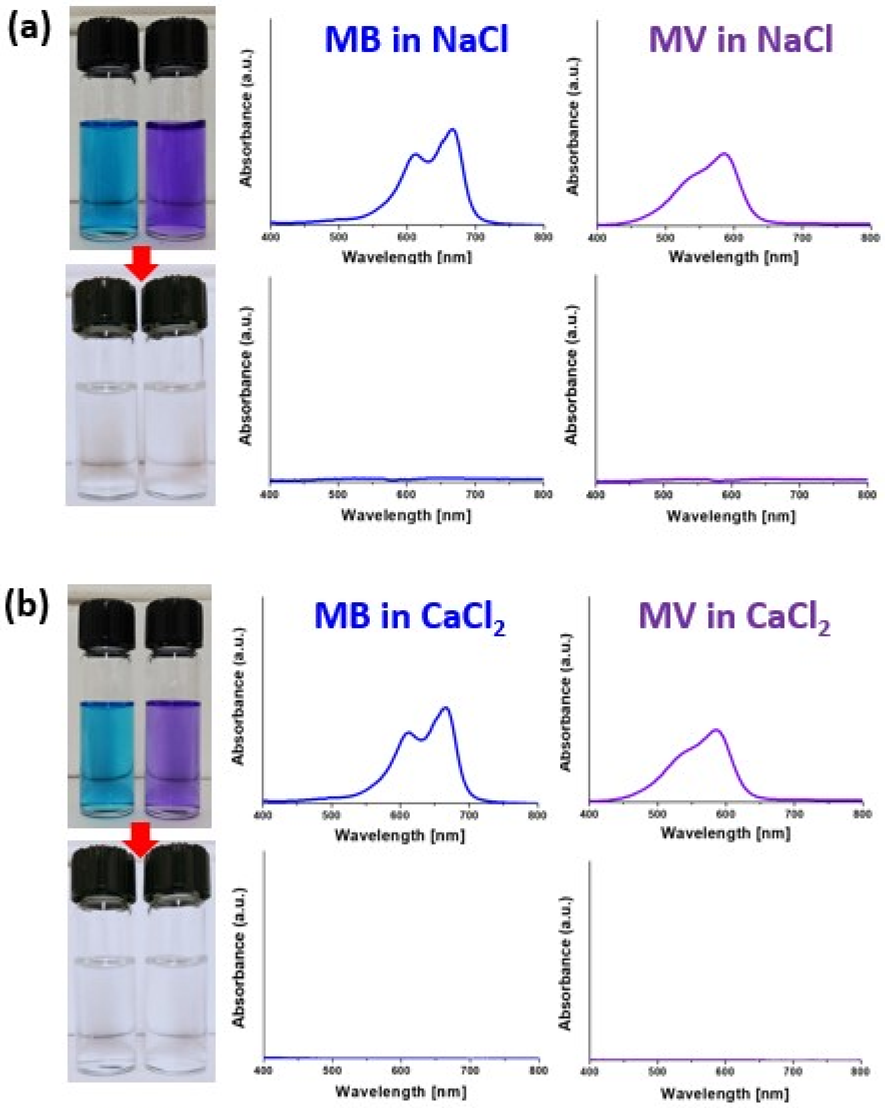

Figure S8. The removal of ~5 ppm of MB or MV by the MXene-*g*-poly(AMPS-*co*-AA) in (a) ~0.3 M NaCl and (b) ~0.3 M $CaCl_2$ solutions, demonstrated by photo images and UV-vis spectroscopy.

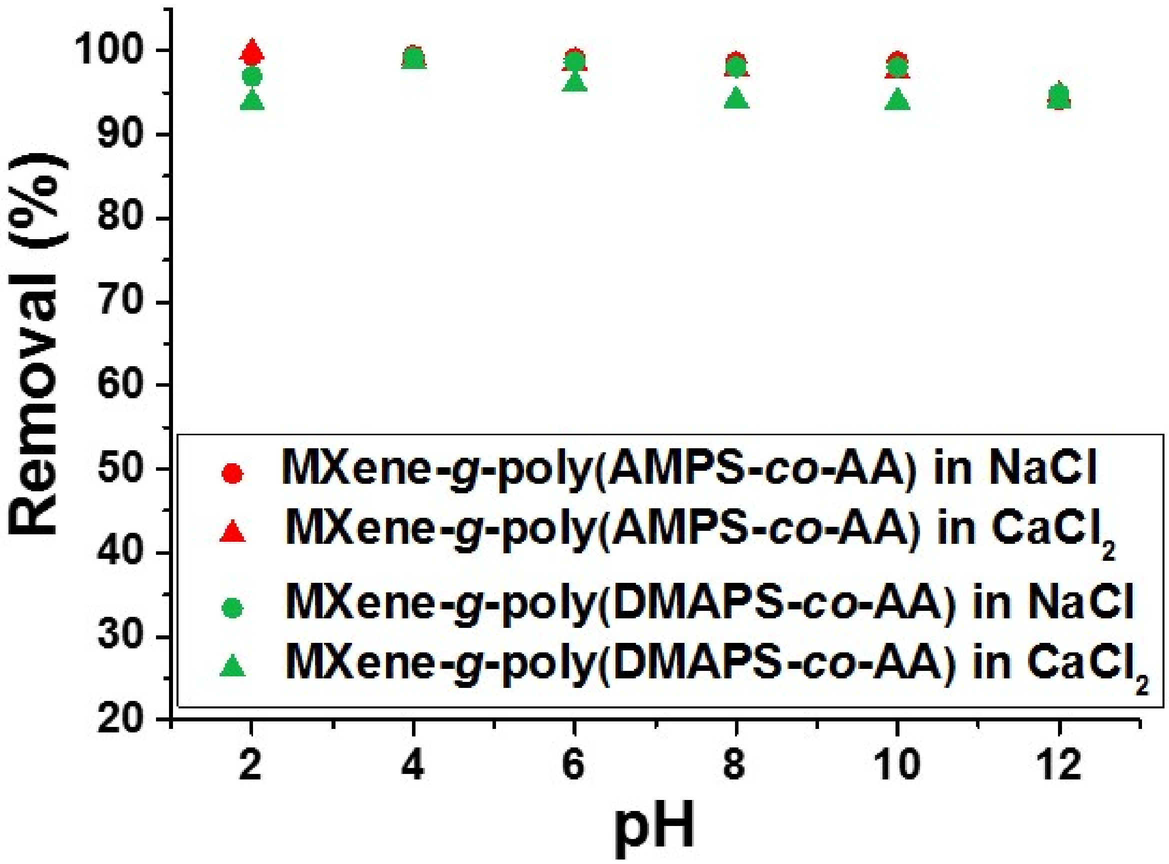

Figure S9. The MB removal efficiencies of the MXene-*g*-poly(AMPS-*co*-AA) (red) and MXene-*g*-poly(DMAPS-*co*-AA) (green) investigated for ~0.3 M NaCl (circles) and ~0.3M $CaCl_2$ solution (triangles) with different pH values (2 – 12) contaminated with ~5 ppm of MB.

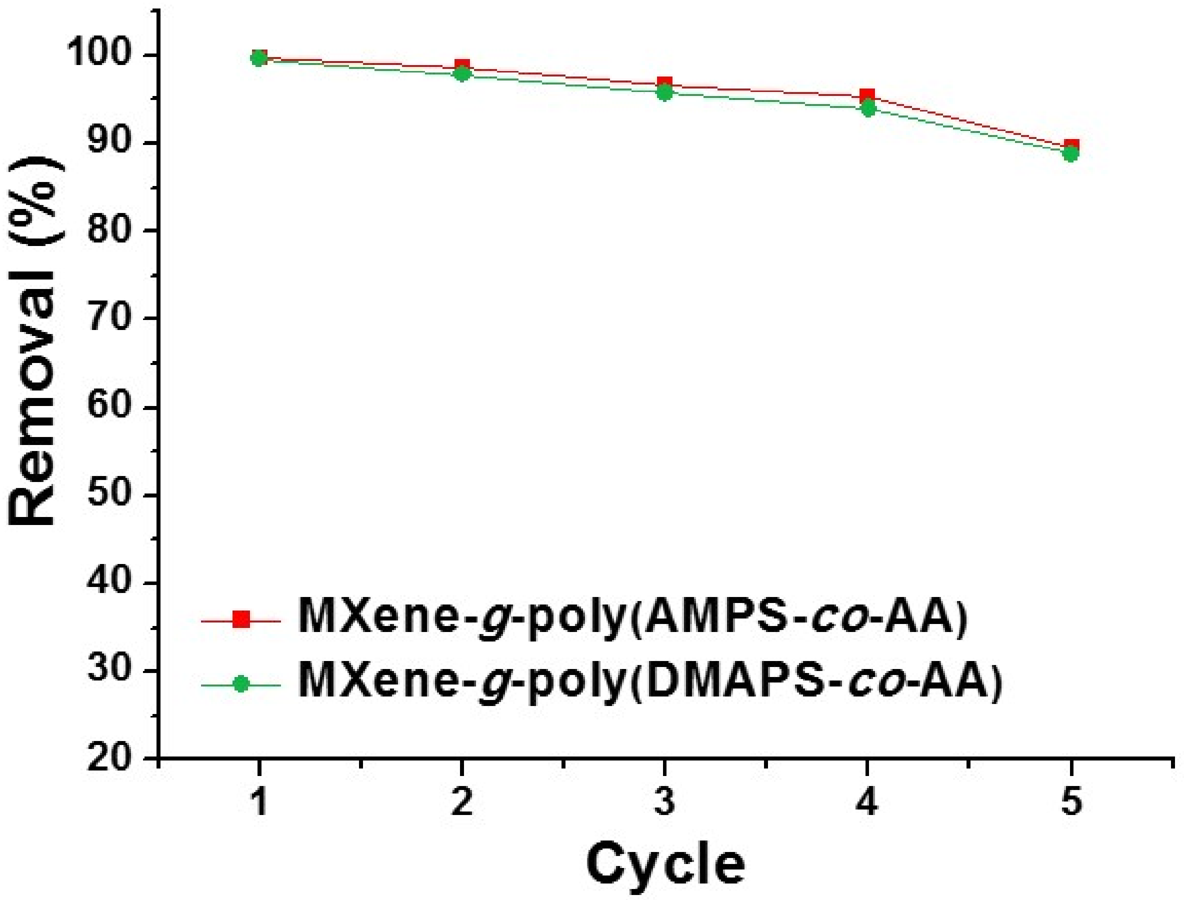

Figure S10. The MB removal efficiencies of the MXene-*g*-poly(AMPS-*co*-AA) (red squares) and MXene-*g*-poly(DMAPS-*co*-AA) (green circles) during five repeated tests performed on ~0.3 M $CaCl_2$ solutions (pH = 8) contaminated with ~5 ppm of MB.
